# Supplementary material for: Whom do we prefer to learn from in observational reinforcement learning?
Source: PLoS Comput Biol. 2025 Dec 8;21(12):e1013143. doi: 10.1371/journal.pcbi.1013143 (PMC12697965; doi:10.1371/journal.pcbi.1013143)
Supplement: S1 Text — (DOCX) [file pcbi.1013143.s001.docx]

**S1 Text. Dynamic-weight model.**

In addition to the computational models reported in the main text, we tested for a dynamic-weight model in which participants initially learn from observed outcomes and later switch to imitation, following previous work [1]. This dynamic-weight model is identical to the full model except that the relative weight of learning from observed actions varies across trials as a function of the reliability of the partner’s action:

$$w_{t}=\frac{1}{1+\exp\left( -\gamma\mathrm{Rel}_{t}+\delta\right)}$$

where Rel_t_ = 1 – PE_t_ is the reliability of the partner’s action, defined as one minus the action prediction error on trial *t*.

We hierarchically fit this model to participants’ choice data in the Observational Learning task. Model parameters *γ* and *δ* were treated as latent variables on the real line (i.e., without transformation). Priors and estimation procedures were specified in the same manner as for the constant-weight full model. Model comparison using WAIC indicated that this new model did not outperform the original constant-weight model (WAIC_dynamic_ – WAIC_constant_ = 4.37), suggesting that participants maintained a stable balance between relying on observed outcomes and imitation across trials.

**References**

1. Charpentier CJ, Wu Q, Min S, Ding W, Cockburn J, and O’Doherty JP. Heterogeneity in strategy use during arbitration between experiential and observational learning. Nature communications 2024; 15:4436.
