# Supplementary material for: Whom do we prefer to learn from in observational reinforcement learning?
Source: PLoS Comput Biol. 2025 Dec 8;21(12):e1013143. doi: 10.1371/journal.pcbi.1013143 (PMC12697965; doi:10.1371/journal.pcbi.1013143)
Supplement: S2 Text — (DOCX) [file pcbi.1013143.s002.docx]

**S2 Text. Correlation between participants' noise level and partner preference.**

We examined whether participants’ own noise levels in the Individual Learning task were correlated with their partner preference. Participants’ noise levels (operationalized as the negative inverse temperature) were estimated using a reinforcement learning (RL) model fit to each participant’s Individual Learning data. This RL model was the same as the one used to derive the three metrics (performance, predictability, and information gain) for the partner preference GLMM reported in the main text.

Partner preference was defined as the proportion of blocks in which participants selected high-noise partners in the Partner Selection task. We then calculated Pearson’s correlation between these two measures. The analysis revealed a positive but non-significant relationship between participants’ noise levels and their preference for high-noise partners (*r*(72) = 0.114 ± 0.116, *p* = .33). The standard error of the correlation was calculated using an approximation formula (1 - *r*²) / √(*n* - 3) [1].

**References**

1. Gnambs T. A brief note on the standard error of the Pearson correlation. Collabra: Psychology 2023; 9:87615.
