# Supplementary material for: Whom do we prefer to learn from in observational reinforcement learning?
Source: PLoS Comput Biol. 2025 Dec 8;21(12):e1013143. doi: 10.1371/journal.pcbi.1013143 (PMC12697965; doi:10.1371/journal.pcbi.1013143)
Supplement: Attachment — Submitted filename: Response to Reviewers.pdf [file pcbi.1013143.s011.pdf]

RESPONSE TO THE REVIEWERS' COMMENTS

OVERVIEW

We thank the reviewers for their thoughtful and constructive feedback. Below we provide a point-by-point response and describe the corresponding revisions to the manuscript (revised text is highlighted in red). In response to the reviewers' comments, we conducted additional data analyses and have revised the text and figures accordingly. We believe these changes substantially strengthen the manuscript and effectively address the reviewers' concerns.

Reviewer #1:

Employing behavioral experiments and computational modelin, this study investigates how people choose whom to learn from in observational learning. The authors tested this question using a pilot and a preregistered experiment and found that people prefer to observe low-noise rather than high-noise individuals. Further analysis suggested that individuals who preferred low-noise models tended to use imitative learning, whereas those who preferred high-noise individuals tended to learn from others' reward outcomes.

The research question is interesting and deserves attention in the field. Although the methods and analyses were conducted rigorously, there are several concerns that need to be addressed:

1. The most important finding in this study is the preference for low-noise demonstrators in observational learning. However, low- and high-noise demonstrators are not clearly distinguished from high- and low-performing demonstrators. Regardless of noise level, choosing better-performing demonstrators is an optimal strategy. Although the authors clearly address this issue in the Discussion, both the title and abstract may mislead readers into interpreting the findings as solely about noise preference rather than performance-based preference.

Reply:

We thank the reviewer for the constructive comments on our manuscript. In response, we have revised the Abstract and Introduction to explicitly state that high-noise demonstrators cannot be distinguished from high- and low-performing demonstrators.

Abstract

(lines 1-13): "*Learning by observing others' experiences is a hallmark of human intelligence. While the neurocomputational mechanisms underlying observational learning are well understood, less is known about whom people prefer to learn from in the context of observational learning. One hypothesis posits that learners prefer individuals who exhibit a high degree of decision noise, 'free riding' on the costly exploration of others. An alternative hypothesis is that learners prefer individuals with low decision noise and tend to imitate consistent and reliable behavior, as lower decision noise is often associated with better performance. In a preregistered experiment, we found that most participants preferred to learn from low-noise (high-performing) individuals. Furthermore, exploratory analyses revealed that participants who preferred low-noise individuals tended to rely on imitation of others' actions. These findings offer a potential computational account of how learning styles are related to partner selection in social learning.*"

Introduction (lines 76-82): "*An alternative possibility is that individuals prefer low-noise partners. Such a preference would be advantageous if they imitate their partners' behavior, since low-noise partners tend to appear more consistent and successful, whereas high-noise partners may be perceived as less competent. Prior work on social learning shows that people are likely to imitate those viewed as more successful [21]. From this perspective, a competing hypothesis is that individuals would prefer low-noise (and thus high-performing) partners.*"

We have retained the original title, as it does not specifically reference noise level or performance. However, we would greatly appreciate any further suggestions from the reviewer, as we remain open to revising the title if necessary.

2. The conclusion that individuals who preferred low-noise demonstrators relied on imitation may require further analysis for confirmation. In a stable environment, once participants identify a demonstrator's performance level, the need for ongoing evaluation may diminish, as they have already determined who is worth learning from. Additionally, the actions of better-performing demonstrators are more consistently tied to better outcomes in later trials, in contrast to the actions of high-noise (or worse-performing) demonstrators. It is therefore possible that participants initially learn from observed outcomes and later switch to imitation, a dynamic not fully captured in the current analyses. This possibility should at least be discussed.

Reply:

Following the reviewer's suggestion, we implemented a dynamic-weight model in which participants initially relied on observed outcomes and gradually transitioned to imitation. Model comparison using WAIC indicated that this new model did not outperform the original constant-weight model, suggesting that participants maintained a stable balance between relying on observed outcomes and imitation across trials. We have added these results to the Results section (see Supporting Information for full details):

Results (lines 242-245): "*Furthermore, we tested for a dynamic-weight model that captures flexible learning styles in which participants initially learn from observed rewards and later switch to imitation (see Methods/SI for details). We found that the model did not outperform the original Full model.*"

3. Does Figure S4 indicate that higher noise levels of the participants themselves in the individual learning task correlate positively (and significantly) with preference for the high-noise demonstrator?

Reply:

Thank you for raising the interesting issue. Figure S4 does not illustrate the relationship between participants' own noise levels in the individual learning task and their preference for high-noise partners. Instead, it presents the parameter estimates of the Full model fitted to the Observational Learning task data, separated by partner preference group.

That said, we also found the relationship between participants' own noise levels in the individual learning task and their partner preference to be a fascinating question. To address this, we conducted an additional data analysis. Specifically, we estimated participants' noise levels in the Individual Learning task and tested the Pearson correlation between these estimates and partner preference. This analysis revealed a weak positive correlation between

preference for high-noise partners and participants' individual noise levels (r(72) = 0.114 ± 0.116). We have added this analysis to Supporting Information as follows.

Supporting Information (lines 17-30): "*We examined whether participants' own noise levels in the Individual Learning task were correlated with their partner preference. Participants' noise levels (operationalized as the negative inverse temperature) were estimated using a reinforcement learning (RL) model fit to each participant's Individual Learning data. This RL model was the same as the one used to derive the three metrics (performance, predictability, and information gain) for the partner-preference GLMM reported in the main text. Partner preference was defined as the proportion of blocks in which participants selected high-noise partners in the Partner Selection task. We then calculated Pearson's correlation between these two measures. The analysis revealed a weak positive relationship between participants' noise levels and their preference for high-noise partners (r(72) = 0.114 ±0.116). The standard error of the correlation was calculated using an approximation formula* $\frac{1-r^2}{\sqrt{n-3}}$ [1]".

Minor points

4. Legend to Figure 1d, last sentence: Please add «of blocks» after «The order» Please check figure numbering in the text throughout (there are for example multiple references to Figure 2 that should refer to Figure 1).

Reply:

We have revised the legend of Fig. 1d accordingly. We also carefully reviewed the manuscript and corrected all figure-number references, including places that mistakenly cited Fig. 2 instead of Fig. 1.

Caption in Figure 1d: "*(d) Timeline of the entire experiment. ... The order of blocks was randomized across participants.*"

Reviewer #2

In this manuscript, the authors investigate two interesting aspects of observational learning (OL): (i) whether people prefer to learn from a high-noise or a low-noise partner after passively observing their performance on a three-armed bandit task, and (ii) whether individual preferences in partner preference relate to observational learning strategy (imitation vs vicarious reward learning) in a subsequent active OL task. They find that overall, participants exhibit a preference for low-noise partners, and that this preference is associated with more imitative learning. These results are interesting and novel, given that partner selection during observational learning has not, to my knowledge, been investigated. I have however, several concerns about the claims related to exploration and am generally unsure if the study design is appropriate for testing the questions of interest.

Major concerns:

1. My main concern, which is raised by the authors during the discussion, but not before, is that there are multiple confounds associated with the noise manipulation (i.e. participants observing and choosing between a low-noise partner and a high-noise partner): cognitive effort (more effort needs to be deployed to learn from a high-noise partner), reward obtained being another one (the low-noise partner performs better), and/or choice reliability/predictability. This makes it virtually impossible to know what factor actually drives partner preference; yet, until the discussion the authors claim that what they manipulate with this is the partner's exploratory tendencies. I think such claim is misleading, and the authors should find a way to at least distinguish exploration from the other confounds (either with additional analyses if possible, or a better designed paradigm). Otherwise, the interpretability of the findings is too low.

In other words, I do believe that there is great premise in operationalizing the preference for low noise vs high exploration (since many real life situations involve this kind of "dilemma"), but I don't think that the current paradigm and analyses tackle this well given that high noise doesn't necessarily mean high exploration. The type of "adventurous" exploratory partner the authors describe is likely rarely random in their exploration; therefore, it would seem key to operationalize an exploratory partner that isn't just driven by high noise, but with directed exploration aiming to maximize information gain.

A potential additional analysis (though I am happy for the authors to find other ways to address the concern) could be to compute behavioral indices of rewards obtained, information gain, action predictability and/or cognitive effort during the passive observation task (maybe extracting those variables from an optimal learner agent since no data is available from the participants during that phase), and use these variables as predictors of partner selection in the first GLMM. This would not only help disentangle potential drivers of partner preference, but also provide some insights into the computational mechanisms involved during passive observation.

Reply:

We thank Dr. Caroline J. Charpentier for the constructive and insightful comments. To address the reviewer's concern, we conducted an additional GLMM analysis to tease out the effects of potential factors underlying the decision noise— "performance", "predictability", and "information gain (i.e., a proxy for directed exploration level)" —on the partner selection.

In the first step of the analysis, we quantified the performance, predictability, and degree of information gain of partners' behavior using the Passive Observation task data.

- The performance was simply calculated by the summation of obtained rewards.
- The perceived predictability was quantified as the extent to which the partner's observed behavior could be predicted from the participant's own decision process. To estimate the decision process the participant would adopt, we fit a reinforcement learning (RL) model with an information bonus (Wilson et al., 2014) to each participant's data from the Individual Learning task. Predictability was then computed as the log likelihood of the partner's behavior in the Passive Observation task, given the participant-specific RL model.
- The perceived information gain was quantified as the extent to which the partner's observed behavior reduced the participant's uncertainty about the available options. To capture this, we used the participant-specific RL model with an information bonus to extract the information bonus parameter. In this model, the information bonus associated with an option decreases as that option is chosen. Information gain was therefore computed as the reduction in information bonus induced by the partner's observed behavior.

For each of the three factors, we computed the difference in the partner-level score (performance, predictability, or information gain) between the two potential partners in the Partner Selection task. We then replaced the partner's noise level dummy variable in the original partner preference GLMM with these three differences. These independent variables were all z-standardized.

The analysis revealed that partner performance was the strongest predictor of partner selection ($b = 0.759 \pm 0.169$; Fig. 2d), followed by predictability ($b = 0.389 \pm 0.161$; Fig. 2d) and information gain ($b = -0.142 \pm 0.193$; Fig. 2d).

In the revised manuscript, we have described the additional data analysis and its results in the Results, Figure 2, Discussion, and Methods sections.

Results: (lines 158-172): "*It is worth noting that decision noise can arise from multiple underlying factors, such as performance and predictability. To examine which factor best accounted for partner selection, we ran an exploratory mixed-effects logistic regression with three predictors: partner performance, predictability, and information gain (a proxy for directed exploration level) estimated from the Passive Observation tasks (see Methods for details). In this exploratory analysis, we combined data from the pilot and main studies, yielding N=74 after excluding one pilot participant who completed only two of the four blocks. We reported effect sizes and standard errors rather than p-values, as p-values are more appropriate for statistical inference based on a priori hypotheses [25, 26] (same for the subsequent analyses). The analysis showed that performance was the strongest positive predictor of partner selection (b=0.759±0.169; Fig. 2d), followed by a weaker effect of predictability (b=0.389±0.161; Fig. 2d). In contrast, information gain had no reliable effect (b=−0.142±0.193; Fig. 2d). Taken together, these results suggest that participants*

preferentially selected low-noise partners primarily because those partners performed better."

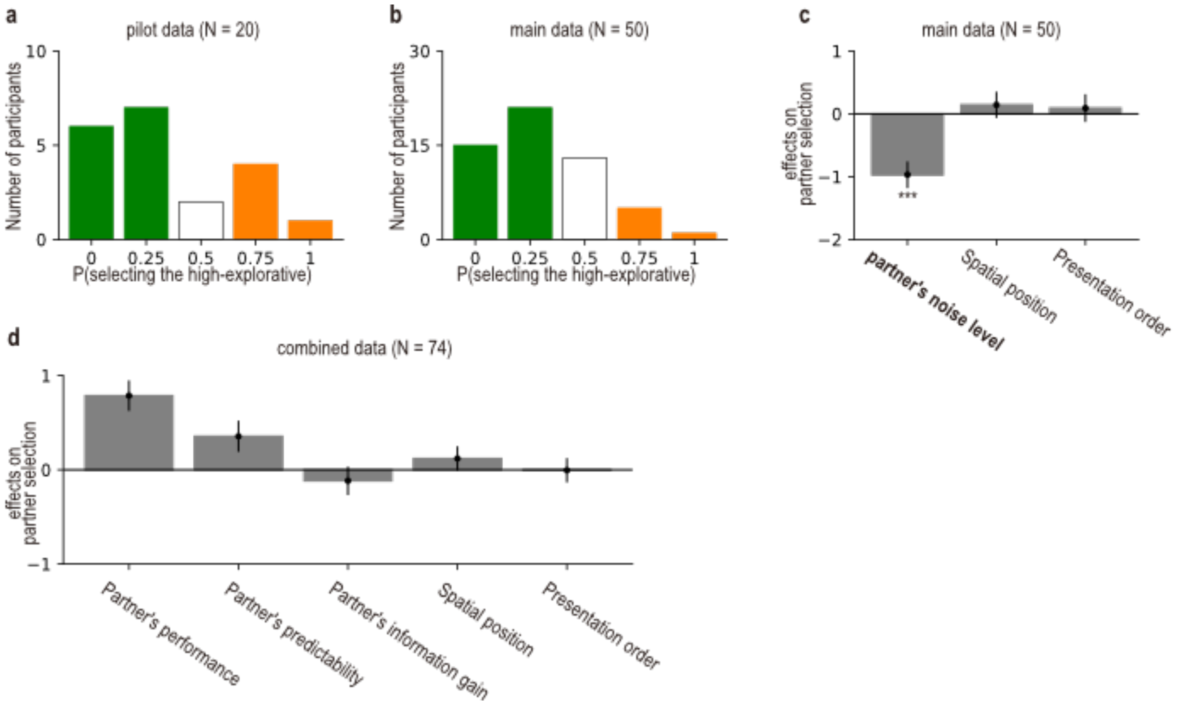

Fig. 2 (a)... (b)… (c)…

(d) Effects on partner selection in the combined data (Mean ± SEM, N = 74). We tested three factors underlying the decision noise manipulation: performance, predictability, and information gain of the partner. P-values are not reported as the analysis is exploratory

Methods (lines596-634): "*We examined whether partner selections were better explained by factors underlying the decision noise manipulation. In the first step of this analysis, we derived three partner-level indices for each potential partner from the Passive Observation data in each block: performance, predictability, and information gain.*

Performance. *We defined performance of the partner as the total obtained reward during the Passive Observation task.*

Predictability. *The perceived predictability was defined as the extent to which the partner's observed behavior could be predicted from the participant's own decision process. To estimate this process, we used a reinforcement learning (RL) model with an information bonus parameter* $\kappa \in [0, 1]$ *[17], such that an information bonus term* $\kappa\frac{1}{\sqrt{N_X}}$ *was added to the action value of option X, where* N_X *denotes the number of times option X had previously been chosen. The RL model was fit hierarchically to each participant's data from the Individual Learning task in the practice block, using the same weakly informative priors and transformation procedure as in the observational learning analysis (see Computational models subsection). Predictability was then quantified as the log likelihood that the participant-specific RL model would generate the partner's observed choices in the Passive Observation task. In other words, higher log likelihood values indicate that the partner's behavior was more consistent and predictable with the decision process estimated for that participant.*

Information gain. *The perceived information gain was defined as the extent to which the partner's observed behavior reduced the participant's uncertainty about the available*

options. To capture this, we used the same RL model with an information bonus parameter. In this model, the information bonus associated with an option decreases by $\kappa(\frac{1}{\sqrt{N_X}} - \frac{1}{\sqrt{N_X+1}})$ *as that option is chosen. Information gain was therefore computed as the total reduction in information bonus induced by the partner's observed behavior.*

For each of the three factors, we subtracted the partner-level score (performance, predictability, or information gain) of the potential partner on the left from that of the potential partner on the right. We then replaced the partner's noise-level dummy variable X_1 *in Model 2 with these three difference scores. All independent variables were z-standardized. In this exploratory regression analysis, we report standardized regression coefficients as effect sizes rather than p-values, as p-values are more appropriate for statistical inferences based on a priori hypotheses [25, 26]. Accordingly, since all subsequent analyses are exploratory, we adopt this reporting practice throughout.*"

Discussion (lines 324-338): "*To disentangle these possibilities, we estimated the effects of three potential factors on partner selection: partner performance, predictability, and information gain (proxy for directed exploration level). The results indicated that partner performance was the strongest predictor of choice, followed by predictability, while the effect of information gain had no reliable effect (Fig. 2d). These findings align with prior studies that people learn more efficiently from high-performing or predictable individuals [14, 32, 33, 34, 35], suggesting that learners preferentially select partners from whom they expect to learn effectively.*

To better tease apart these influences, future studies should experimentally decouple exploration from performance and predictability. One approach would be to design tasks in which partners with different levels of exploration nonetheless achieve comparable accuracy and predictability, for example, by manipulating the degree of directed exploration so that both high- and low-explorative partners obtain similar amounts of rewards. Such designs would more cleanly isolate the unique effect of exploration on partner selection."

Furthermore, we have revised the Introduction to downplay the emphasis on equating decision noise with exploration and to articulate the alternative account (e.g., preference could be driven by other factors such as performance/predictability).

Introduction (lines 76-82): "*An alternative possibility is that individuals prefer low-noise partners. Such a preference would be advantageous if they imitate their partners' behavior, since low-noise partners tend to appear more consistent and successful, whereas high-noise partners may be perceived as less competent. Prior work on social learning shows that people tend to imitate those viewed as more successful [21]. From this perspective, a competing hypothesis is that individuals would prefer low-noise (and thus high-performing) partners.*"

2. The authors' initial hypothesis about the individual differences "when individuals primarily rely on imitation, they may show a preference for low noise partners". Yet, individuals' OL style is not assessed before they are asked to choose a partner. It would be helpful to further justify why the study was designed that way

(i.e. without a possibility to assess participants' learning style at baseline). While this study is not designed to test causality, the hypothesis above is qualitatively different from the finding that "individuals who preferred low-noise partners demonstrated a greater reliance on imitative learning".

Reply:

We apologize that the original statement, "when individuals primarily rely on imitation, they may show a preference for low-noise partners," was misleading. Our intention was not to suggest that a stable personal trait (i.e., a general tendency to imitate) determines the preference for low-noise partners. Rather, we aimed to describe a correlational relationship between reliance on imitation and partner preference, which is why we did not include a separate baseline task to assess participants' observational learning style prior to the partner-choice phase. We have revised the Introduction section to clarify this point (see the response to Comment #1).

3. More generally, it would be interesting to further discuss whether both learning style and partner preferences are likely to vary depending on the context (and thus, it's likely because participants chose a low-noise partner that they rely more on imitation), or whether they are more stable and trait-like. Maybe some exploratory behavioral analyses on participants who show some variability in their partner selection across blocks could speak to that? For example, do the same participants exhibit higher partner's action effect/lower partner's reward effect in the blocks where they chose the low-noise partner compared to blocks where they chose the high-noise partner? If so it might be interesting to test a computational model in which the weight parameter varies depending on the chosen partner.

Reply:

Thank you for raising the fascinating issue. We examined whether participants adjusted their observational-learning style depending on the partner type. To this end, we have conducted a within-participant regression analysis focusing on the data from participants who observed both the low- and high-noise partners. The additional data analysis showed evidence for the contextual modulation: i.e., the same participants exhibited a higher action (imitation) effect ($b = -0.355 \pm 0.038$, N=52; Fig. 4a), and a slightly lower reward effect ($b = -0.136 \pm 0.046$, N=52; Fig. 4a) when observing a low-noise partner compared to a high-noise partner. In the revised manuscript, we have reported these findings as follows.

Results (lines 258-268): "*Finally, we examined whether and how participants adjusted their learning style according to the partner's noise level (i.e., a within-participant effect). To this end, we analyzed trial-by-trial behavior in the Observational Learning task among participants who selected both high- and low-noise partners at least once (N=52). This additional GLMM analysis revealed that the reward effect was attenuated when participants observed a high-noise partner ($b=-0.113\pm0.025$; Fig. 4). More critically, the influence of the partner's actions on participants' choices varied markedly with the partner's noise level, as reflected by a strong negative interaction ($b=-0.375\pm0.021$; Fig. 4). This pattern indicates that the same participants relied more heavily on their partner's actions when the partner was low-noise, and suppressed imitation when the partner was high-noise.*"

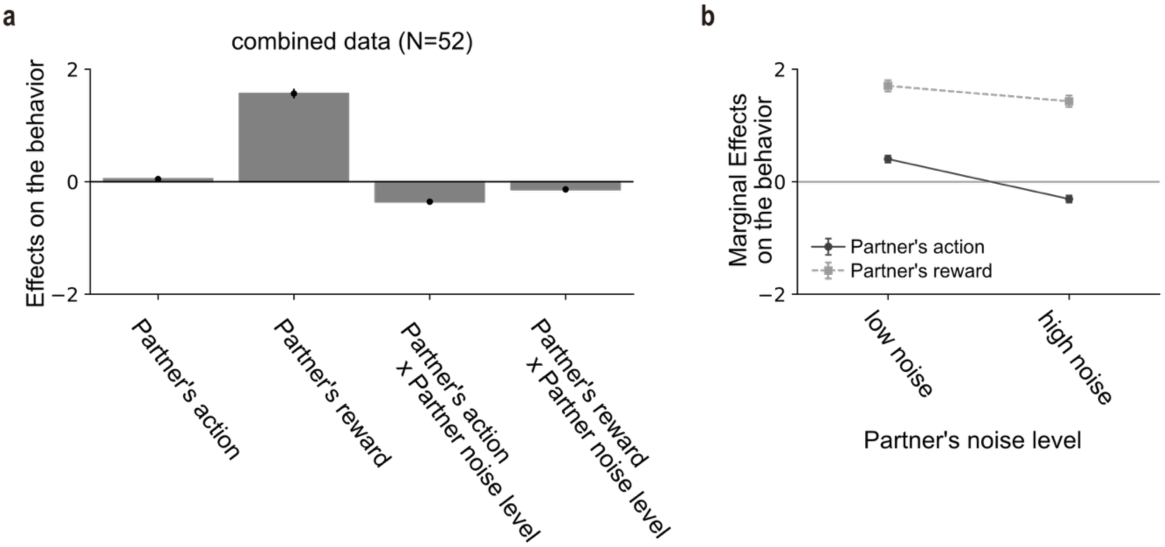

Figure 4: Exploratory analyses of within-participant changes in learning style by the partner's noise level (N=52 participants who selected both high- and low-noise partners at least once). (a) Effects on participants' trial-by-trial behavior in the Observational Learning task (mean ± SEM). The first and second bars represent the main effects of the partner's action and reward, respectively, while the third bar shows the interaction between the partner's action and the partner's noise level. Means and SEMs were estimated using a GLMM. P-values are not reported as the regression analysis was exploratory. (b) Marginal effects (simple slopes) from the GLMM. Effects of the partner's action (solid line) and the partner's reward (dashed line) are shown at two different levels of partner decision noise.

Discussion (lines 360-377): "*However, this analysis cannot determine whether individual differences in learning style reflect partner preferences or are induced by differences in the partner's noise level. Two possibilities remain. First, a stable tendency to imitate may drive the preference for low-noise partners. Second, the behavior of a low-noise (high-performing) partner may shift participants toward imitation more than the behavior of a high-noise (low-performing) partner.*

To distinguish between these two accounts, we tested whether participants' learning style changed with the noise level of the selected partner within a participant. Restricting the analysis to participants who selected both high- and low-noise partners at least once, we estimated within-participant changes in learning style. Participants showed greater reliance on imitation when observing low-noise partners, consistent with prior studies showing selective imitation of low-noise (high-performing) partners [32, 33, 34, 35]. This finding supports the second explanation: the behavior of a low-noise (high-performing) partner may shift participants toward imitation. However, this approach has an important limitation: because it requires exposure to both high- and low-noise partners, it includes only a subset of participants, raising the possibility of selection bias. To address this issue more directly, future work should systematically manipulate partner noise levels within participants."

4. Given that the 0.25, 0.5, 0.75 probabilistic structure was the same across both passive and active tasks, is it possible that participants could have learned by mapping that structure across the two tasks (even with the stimuli being different)? Was the position of the 0.25, 0.5 and 0.75 stimuli different between the blocks? It would be helpful to mention this probabilistic structure in Figure 1 and/or in the overview of the experimental design, since this is important information.

Reply:

The mapping between reward probabilities and screen positions was randomized across both tasks as well as across blocks, preventing participants from directly transferring the probabilistic structure between tasks. We have clarified this point in the revised manuscript as follows.

Revisions:

Results (lines 118-121): "*Importantly, the set of reward probabilities (0.25, 0.50, and 0.75) was identical in both the Passive Observation task and the subsequent Observational Learning task. However, the fractal stimuli and screen positions associated with these probabilities were randomized across tasks and across blocks.*"

5. In addition to picking the best model and comparing the parameter values, it would further strengthen the claim if the authors could show that participants who prefer the low-noise partner (from Figure 2a-b) are best fit by the action learning model, while participants who prefer the high-noise partner are best fit by the reward learning model.

Reply:

Thank you for the constructive suggestion. We conducted model comparisons separately for participants who preferred low-noise partners and those who preferred high-noise partners (as shown in Fig. 2a–b). Specifically, we repeated the hierarchical model comparison within each subgroup. In both cases, the Full model provided the best fit according to WAIC. We have reported these findings in the Results section and Supporting Information.

Results (lines 239-241): "*We also conducted model comparisons separately for participants who preferred low-noise versus high-noise partners, and confirmed that the Full model provided the best fit in both of the groups (Fig. S6).*"

Supporting Information:

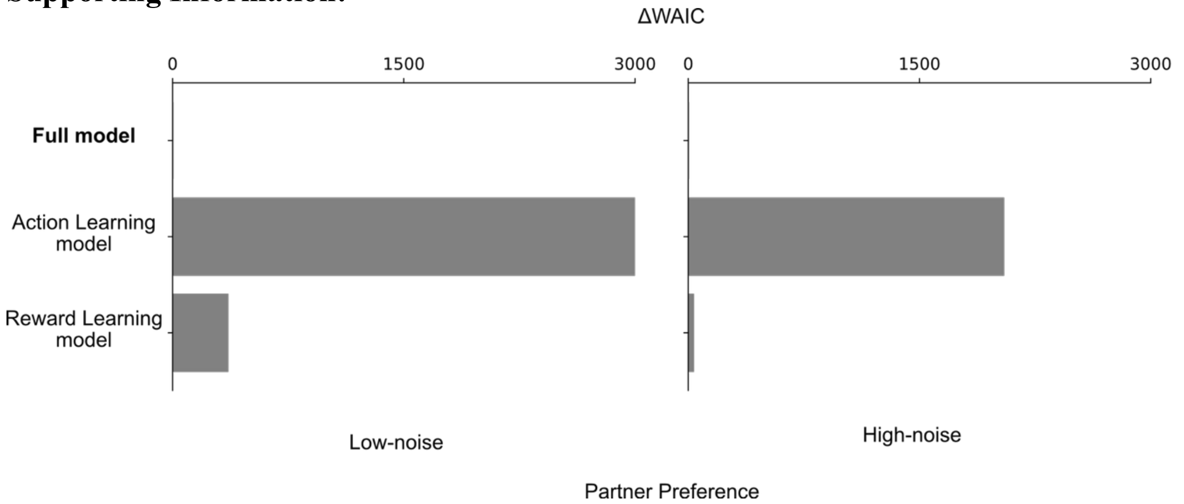

Figure S6: Model comparison stratified by partner preference. We conducted model comparisons separately for participants who preferred low-noise partners and those who preferred high-noise partners (Fig. 2a–b in the main text). The Full model provided the best fit according to WAIC in both of the groups.

6. While it was great to see robust parameter and model recovery, it would also be great if the authors could perform posterior predictive checks to ensure the model captures behavioral tendencies. This could include reproducing the learning curves from model-generated data, and reproducing the GLMM effects from Figure 3a.

Reply:

Thank you for the helpful suggestion. We have performed the posterior predictive checks. The results replicate the observed pattern in the actual data about the learning curves and GLMM effects. Please see Fig. S5 in the revised manuscript as well as the Methods section (please see Revision section below).

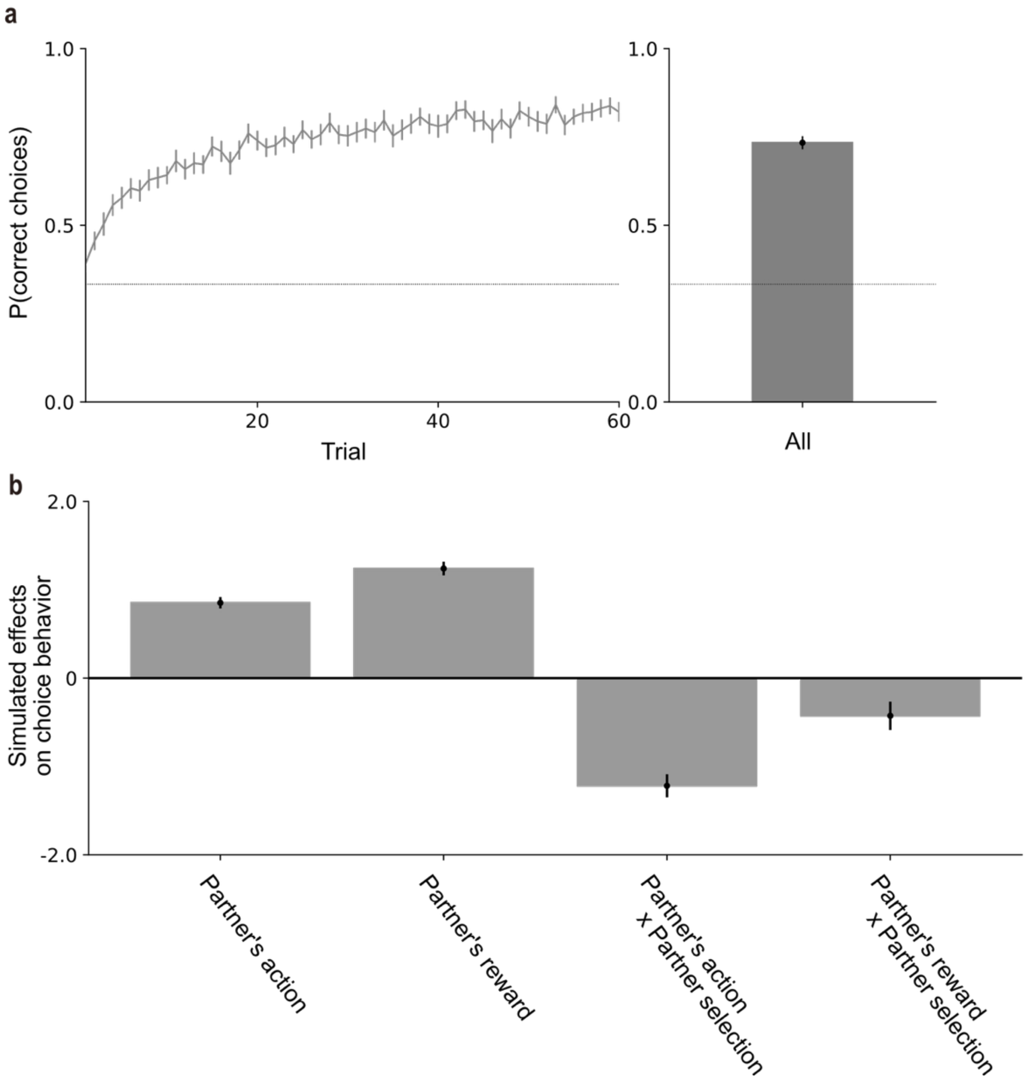

Figure S5: Posterior predictive checks. (a) Simulated learning curve and overall accuracy in the Observational Learning task (Fig. S1c). Left panel: the simulated proportion of correct choices (mean across 4 blocks ± SEMs). Right panel: simulated overall accuracy (Mean ± SEM) (b) Simulated effects of partner-related variables on participants' behavior (Mean ± SEM). We re-fit the same generalized linear mixed-effects model (GLMM) 500 times, obtaining the means and SEMs of simulated effects.

Results (lines 232-236): "*We also conducted posterior predictive checks to examine whether the Full model could reproduce the empirical behavioral patterns (i.e., the learning curve (Fig. S1c) and the effects of observed reward and action on the behavior in the Observational*

Learning tasks (Fig. 3a)), supporting that the model captures key behavioral tendencies (Fig. S5). These simulation results support the validity of our model-fitting procedure."

Methods (lines 703-713): "*We conducted posterior predictive checks to assess whether the Full model could reproduce key behavioral patterns. Specifically, we examined whether model-generated choices recovered the learning curve (Fig. S1c) and the effects of the partner's actions and rewards on the participant's behavior in the Observational Learning task (Fig. 3a). To this end, we drew individual parameter estimates for all 74 participants from the fitted Full model and simulated choice data for four blocks of the task under the same empirical conditions (trial structure, reward contingencies, and partner noise level). We then computed the learning curve averaged across participants (Fig. S5a). In addition, we fitted the simulated choice data using the same GLMM applied in the empirical data analysis (Fig. 3a). Repeating this procedure 500 times yielded the means and standard deviations of the fixed-effect estimates (Fig. S5b).***"**

7. I assume it is more difficult for the model to separate the two learning strategies when it's learning from the low-noise partner (because the action and reward are confounded on a higher proportion of trial). Is this true? If so, could that be impacting the individual difference results in any way?

Reply:

To address this issue, we examined whether differences in partner noise level preference could affect parameter recoverability of the Full model by conducting a group-stratified assessment. Specifically, we repeated the parameter recovery procedure, splitting simulated participants into two groups based on partner preference, and then calculated correlations between true and estimated parameters. The results indicated good recoverability in both groups.

For additional robustness, we also computed histograms of bias (true – estimated) for each model parameter, stratified by partner preference. This analysis likewise showed no systematic bias.

Together, these results suggest that parameter recoverability did not differ between noise levels of the partners, and thus the individual-difference results are unlikely to be affected by such effects.

We have revised the manuscript to add this analysis as follows.

Methods (**lines 692-701**): "*To test whether differences in partner noise-level preference could affect parameter recoverability of the Full model, we also conducted a group-stratified assessment. Simulated participants were divided into high-noise preference (choosing high-noise partners more than twice) and low-noise preference (choosing low-noise partners more than twice) groups, discarding no-preference participants (choosing high- and low-noise partners equally). We computed the same parameter-wise Pearson correlations between true and estimated values, and additionally examined the distribution of signed estimation errors separately within each group (Fig. S2c). Strong positive correlations in both groups, coupled with estimation error distributions centered near zero, indicate that parameter recovery works equally well across the two noise-level preference groups.*"

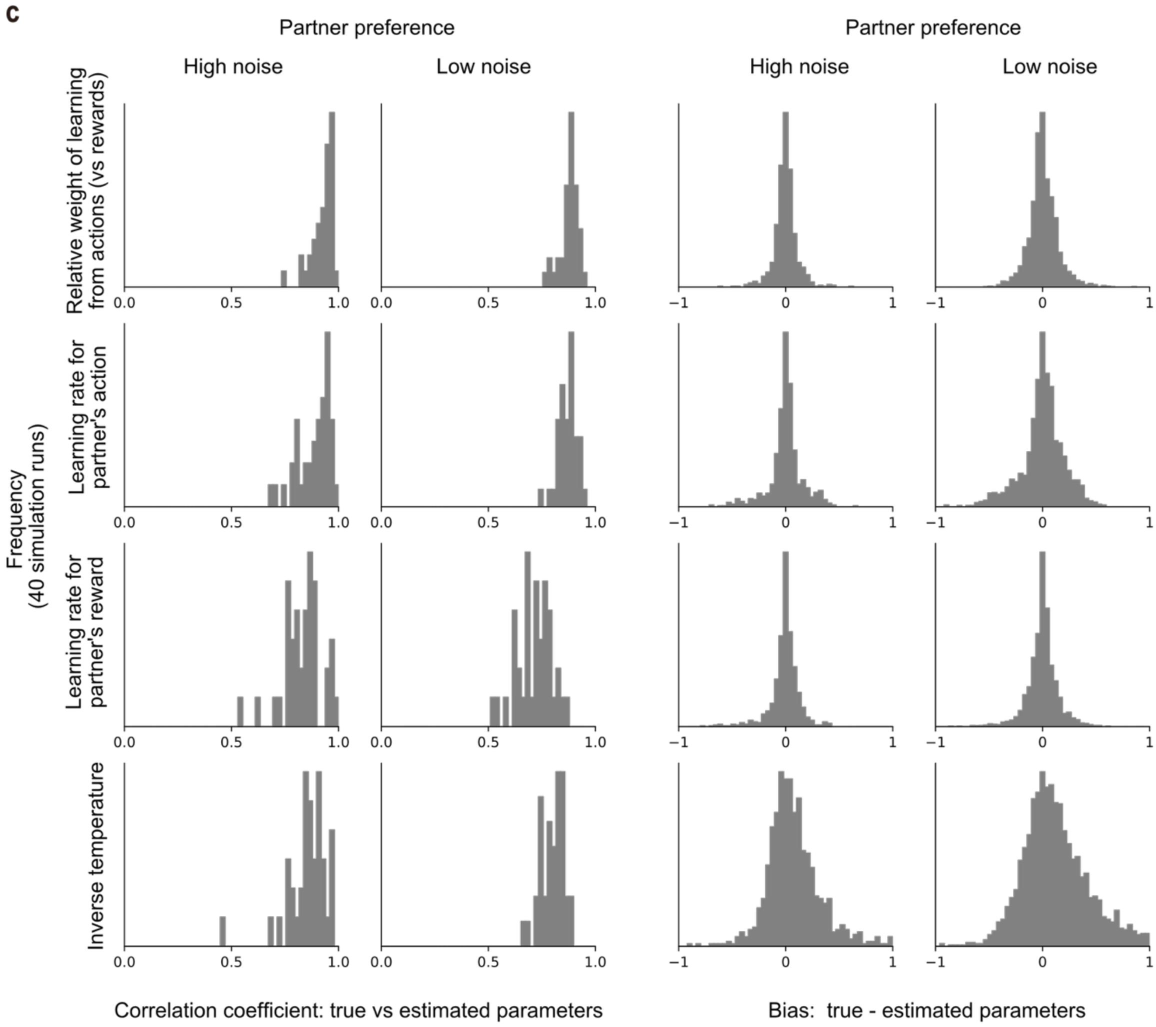

Fig. S2 (c): Distribution of parameter-recovery correlations (left) and biases (right) stratified by noise-preference group. Each row corresponds to one recovered parameter: the relative weight of learning from the partner's action, the learning rate for the partner's action, the learning rate for the partner's reward, and the inverse temperature. For correlations (two left columns), each histogram shows the distribution of Pearson correlation coefficients between true and estimated parameters across 40 simulated datasets, separately for high-noise preference and low-noise preference groups. For biases (two right columns), each histogram shows the distribution of signed estimation errors (estimated - true) under the same stratification. Correlation distributions are strongly shifted toward positive values in both groups, and bias distributions are tightly centered around zero, indicating reliable recovery without systematic over- or under-estimation in either stratum.

Minor comments:

8. It would be helpful if the legend of Figure 1 could mention the number of trials in each task.

Reply:

Thanks for the suggestion. We have added the number of trials for each task to Figure 1 and its caption.

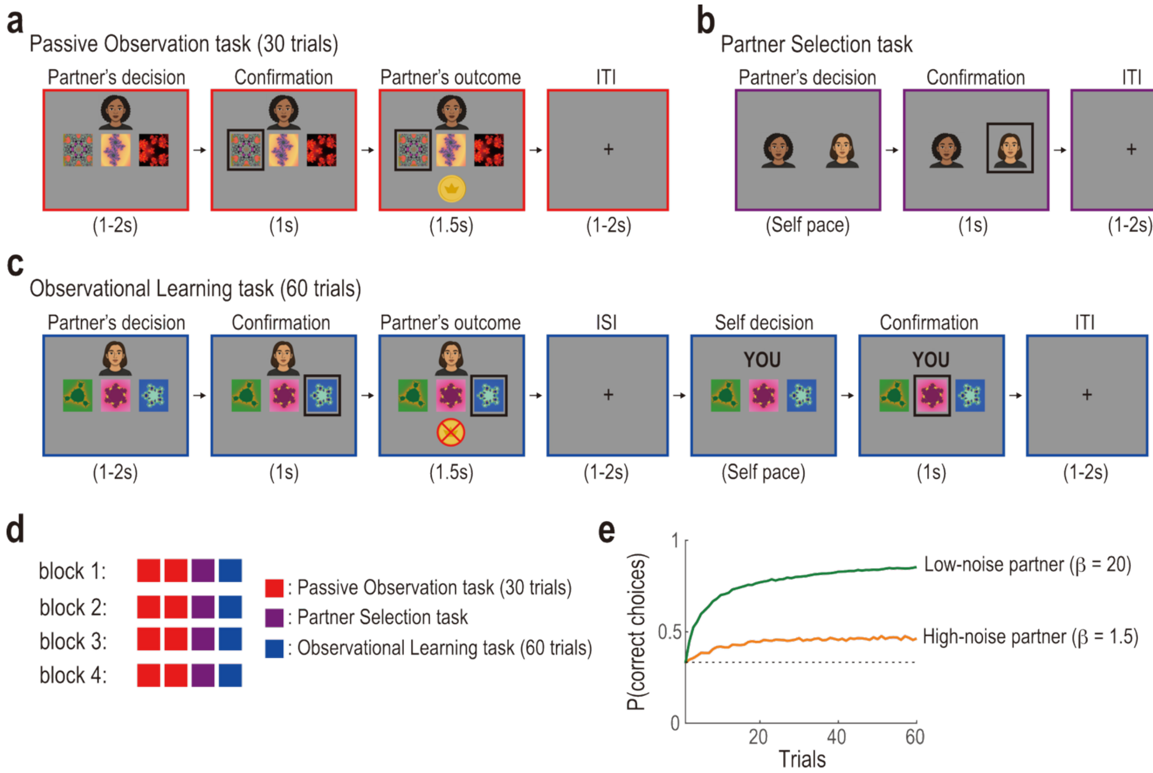

Figure 1: (a) Timeline of each trial in the Passive Observation task. Participants observed a potential partner choose between three options and receive a reward or not over 30 trials. (b) Partner Selection task. Participants chose between two potential partners they had observed in the Passive Observation task as the partner with whom they would perform the Observational Learning task. (c) Timeline of each trial in the Observational Learning task. Participants observed the partner's choice and outcome, and then made their own choice between the same set of options, without observing their own outcome over 60 trials. …

9. Was the presentation order of the two potential partners during the passive observation task also counterbalanced across blocks within participants? According to the methods, it seems like this was counterbalanced across blocks but this wasn't made clear when first describing the task.

Reply:

We apologize for the lack of clarity in our original description. Yes, the presentation order of the two potential partners was also counterbalanced across the tasks and across blocks within participants. We have clarified this in the main text.

Results (lines 104-110): "*In each of the two Passive Observation tasks, participants observed one potential partner performing a three-armed bandit task (i.e., repeatedly choosing one of the three options and receiving a reward based on the probability associated with the chosen option; Fig. 1a) [22, 23, 24]. The potential partner differed between the first and second Passive Observation tasks: one demonstrated a high degree of decision noise, while the other exhibited a low degree of decision noise (Fig. 1e). The presentation order of the two types of potential partners was counterbalanced across the two Passive Observation tasks and across the four main blocks.*"

10. Basic behavior: please also report the proportion and correct choices and learning curves for the main OL task (Figure S1c) broken down by blocks and/ or by selected partner for completeness.

Reply:

Thank you for the suggestion. We have added learning curves broken down by block and by partner preference to Fig. S1 as follows:

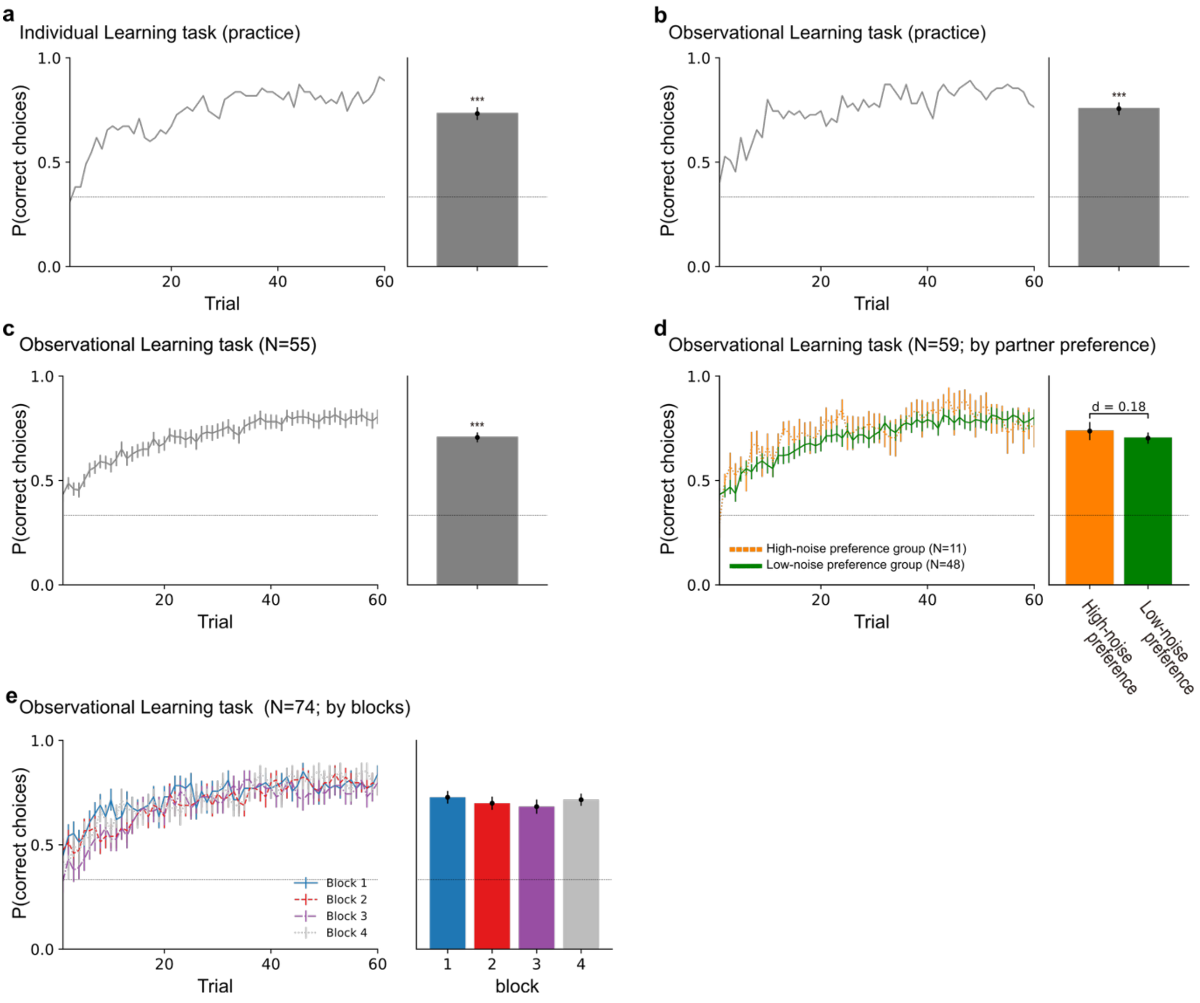

Fig. S1 Each panel contains two plots: on the left, the proportion of correct choices across trials (chance level = 1/3); on the right, overall accuracy (mean ± SEM).

(a) …(b) …(c) … (d) Observational Learning (main + pilot), stratified by partner preference. High-noise (orange dashed) vs. low-noise (green solid). (e) Observational Learning (main + pilot), stratified by block. Results for Blocks 1–4 (distinct colors).

11. Figure 3. For illustrative purposes, it would be helpful to extract and plot the marginal means of the two interaction effects from the GLMM in panel a. This would allow the reader to visualize the effects of partner's action and partner's reward separately for each of the 5 partner selection levels.

Reply:

Thanks for the helpful suggestion. We now plot the marginal effects (simple slopes) from the GLMM in Fig. 3b. Specifically, we show the estimated effects of the partner's action and reward on choice at five levels of partner selection (0, 0.25, 0.5, 0.75, 1; defined as each participant's proportion of blocks choosing the high-noise partner).

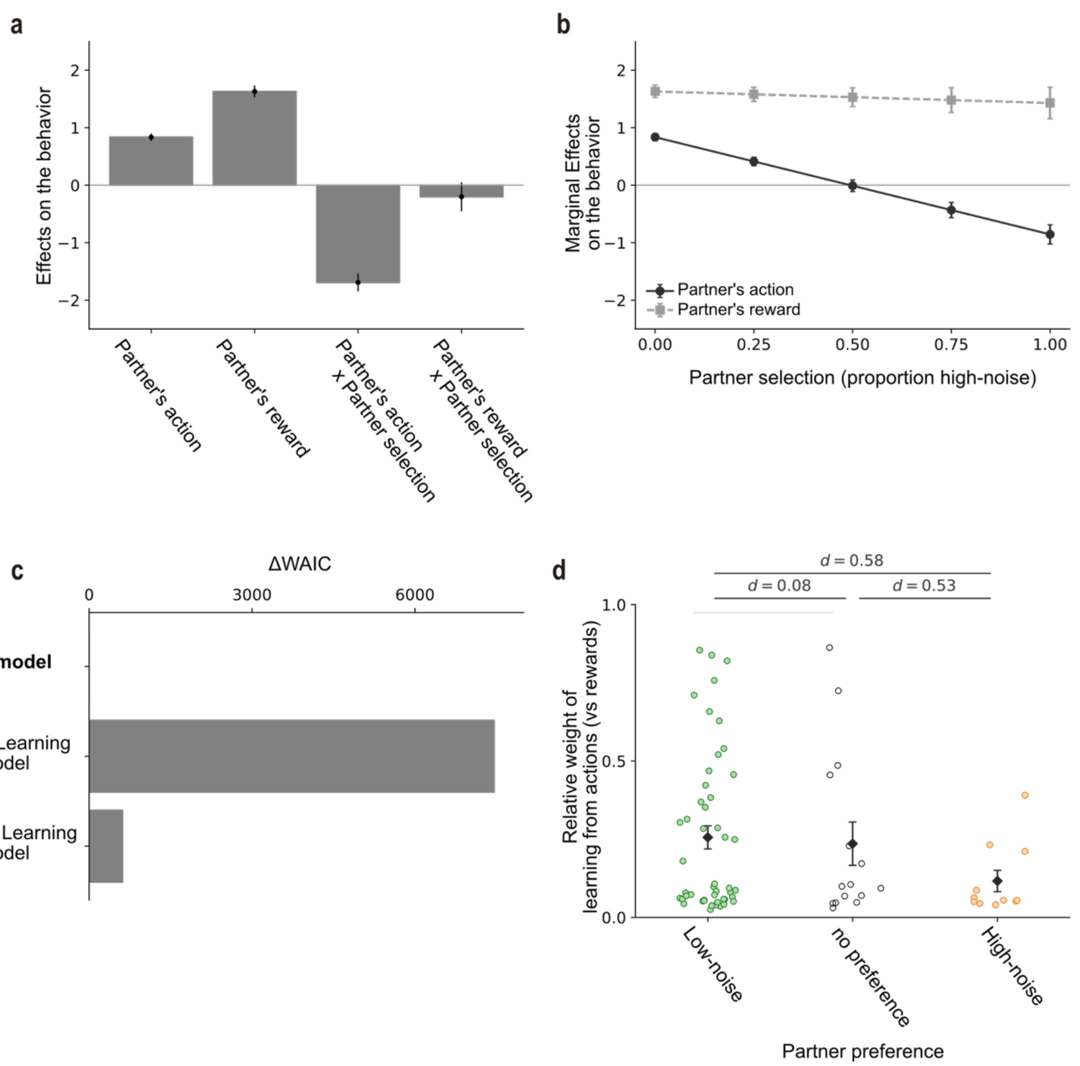

Figure 3*: Exploratory analyses of individual learning styles. (a) …(b) Marginal effects (simple slopes) from the GLMM. Effects of the partner's action (solid) and partner's reward (dashed) evaluated at five levels of high-noise preference. Error bars show ± SEM.* (c)… (d)…

12. Figure 3c. Is this effect specific to the action learning weight? Is it possible that participants in the low-noise partner selection group are simply better learners and therefore also show better performance and better reward learning? I understand from Figure S4 that other parameters do not show the same group differences; however, it would be helpful to also compare behavioral metrics such as overall performance or learning curves.

Reply:

Thank you for the helpful suggestion. To examine whether the finding in Fig. 3c (now Fig. 3d) reflects action learning specifically rather than a general performance advantage, we compared overall performance and learning curves between the two partner-preference groups (Fig. S1d). We have added this figure to the Supplementary Information and report the corresponding effect size (d = 0.18). The results did not provide strong evidence that one

group consistently outperformed the other.

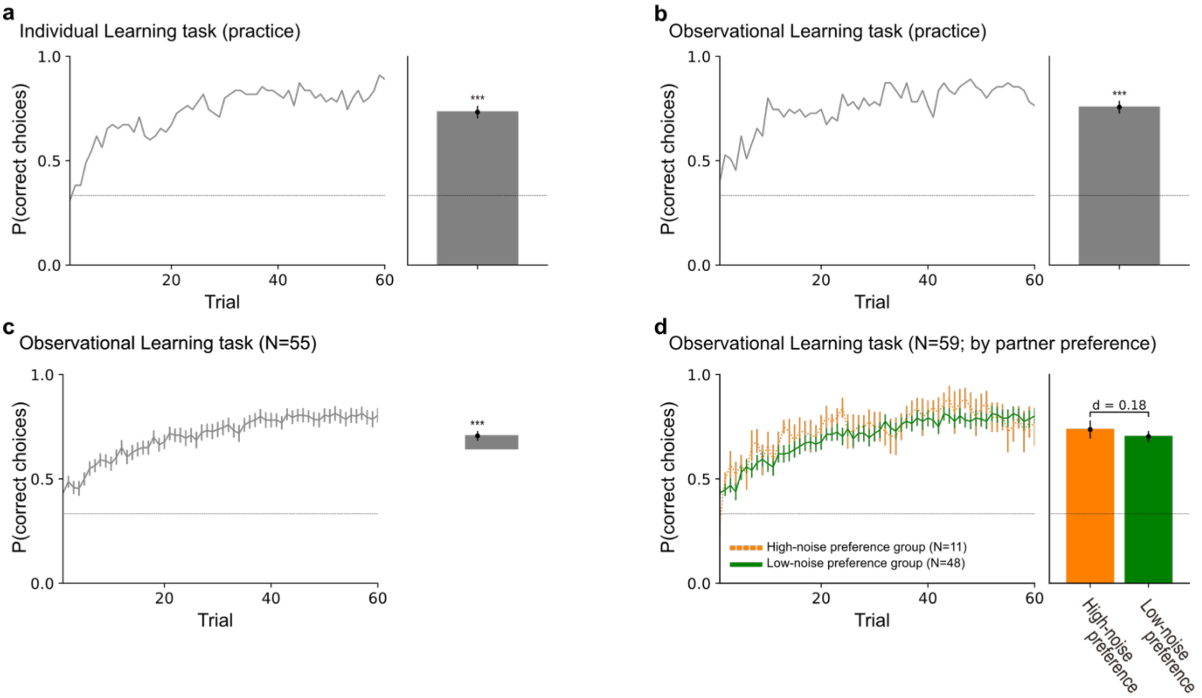

Figure S1. The proportions of correct choices.

(a)… (b)… (c)…(d) Observational Learning (main + pilot), stratified by partner–noise preference. High-noise (orange dashed) vs. low-noise (green solid). (e)…

13. Since there is only one weight parameter in the model it would be helpful to rename Figure 3c y-axis with something that reflects that this is not just the weight of learning from the partner's action but the relative weight of learning from actions versus rewards. Same comment for Fig S2b title of left panel.

Reply:

Thank you for the helpful suggestion. We have revised the labeling to make the relative nature explicit: the y-axis in Fig. 3d (formerly Fig. 3c) now reads "Relative weight of learning from actions (vs rewards)", and we applied the same wording to the title of the left panel in Fig. S2b.

d

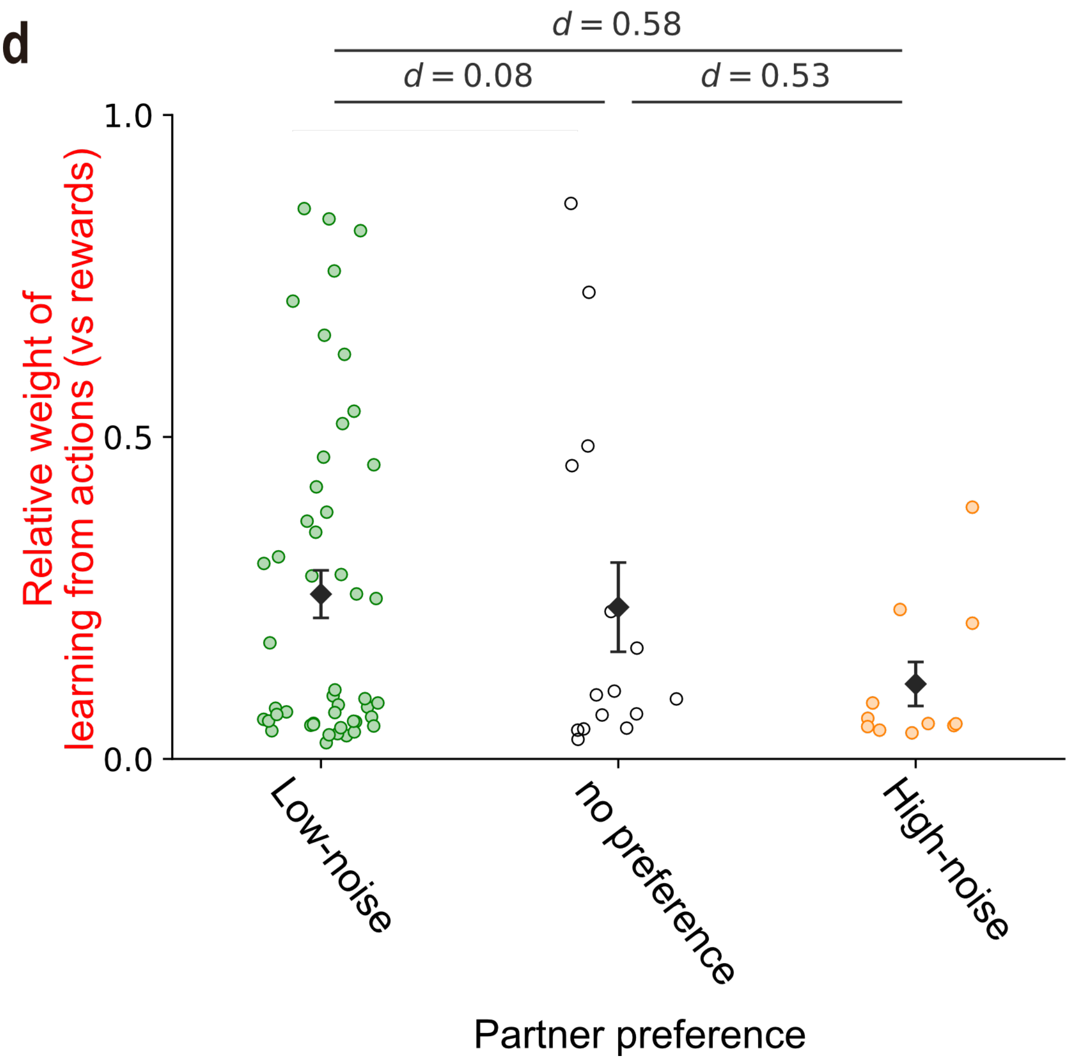

d = 0.58
d = 0.08
d = 0.53
1.0
0.5
0.0
Relative weight of learning from actions (vs rewards)
Low-noise
no preference
High-noise
Partner preference

b

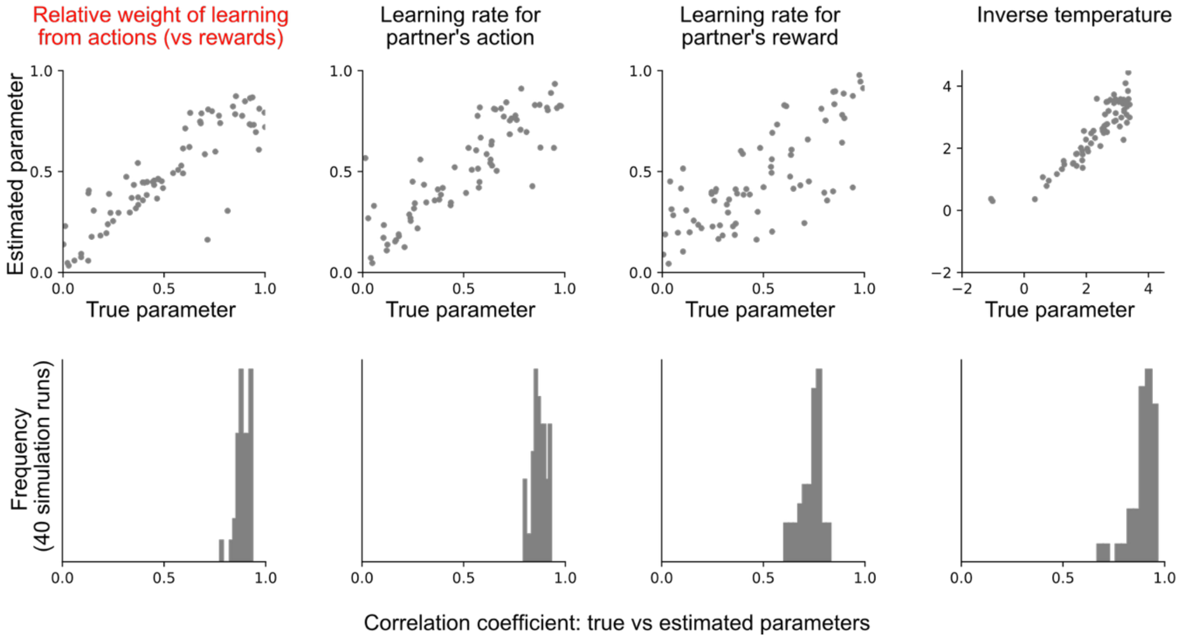

Relative weight of learning from actions (vs rewards)
Learning rate for partner's action
Learning rate for partner's reward
Inverse temperature
Estimated parameter
True parameter
True parameter
True parameter
True parameter
Frequency (40 simulation runs)
Correlation coefficient: true vs estimated parameters

14. Model/parameter recovery (Figure S2) why was the number of simulations different for the two analyses (30 vs 40 simulations). The y-axis title of Fig S2b bottom row suggests that parameter recovery was also 30 simulations, which is inconsistent with the legend and methods.

Reply:

The original difference (30 vs. 40) reflected initial runtime constraints. To eliminate confusion, we reran the model recovery with 10 additional replicates so that both model and parameter recovery now use 40 simulations per condition. We have updated the Methods, the Fig. S2 legend, and the y-axis title of Fig. S2b (bottom row) accordingly. The expanded runs yielded virtually identical patterns and did not change any conclusions.

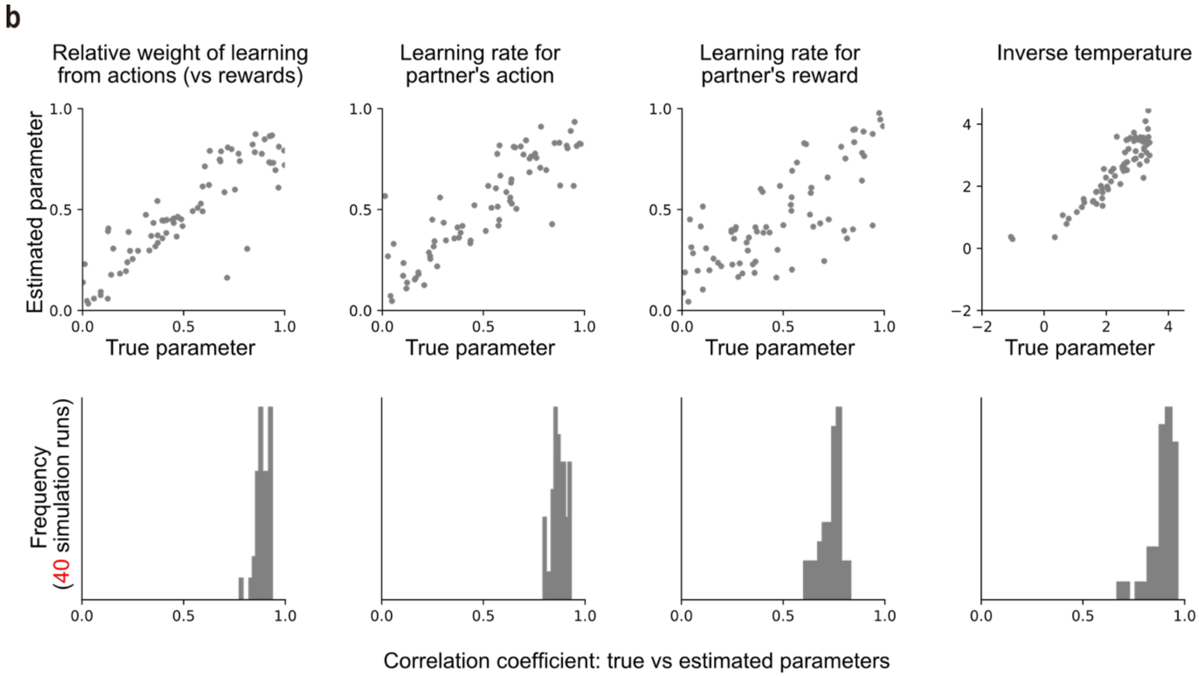

15. Figure S2a. Please write the exact numbers on the matrix as it is difficult to match them to the color scale. Especially the action learning model seems less well recovered than the other two but it is unclear what it might be confused with.

Reply

Thank you for the helpful suggestion. We have revised Fig. S2a to overlay exact counts and row-wise proportions in each cell (e.g., “35 (0.88)”), so the confusion matrix is readable without relying on the color scale.

a

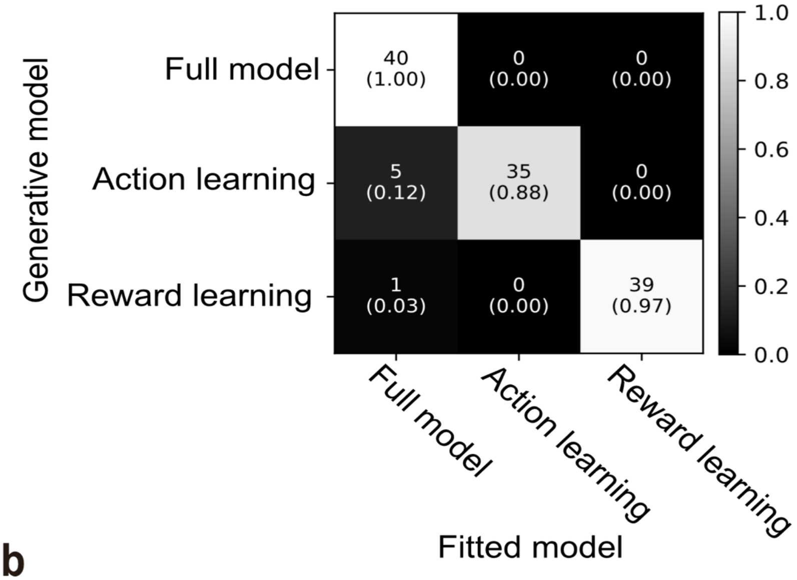

b

16. For the plots in Figure 3c and Figure S4, it would be helpful to depict the mean value of the parameter (or mean of the posterior) so it's easier to visualize the direction of the differences between groups.

Reply:

Thank you for the helpful suggestion. We have now overlaid group means on the scatterplots in Fig. 3d (formerly 3c) and Fig. S4: black diamonds mark the mean with ±SE error bars.

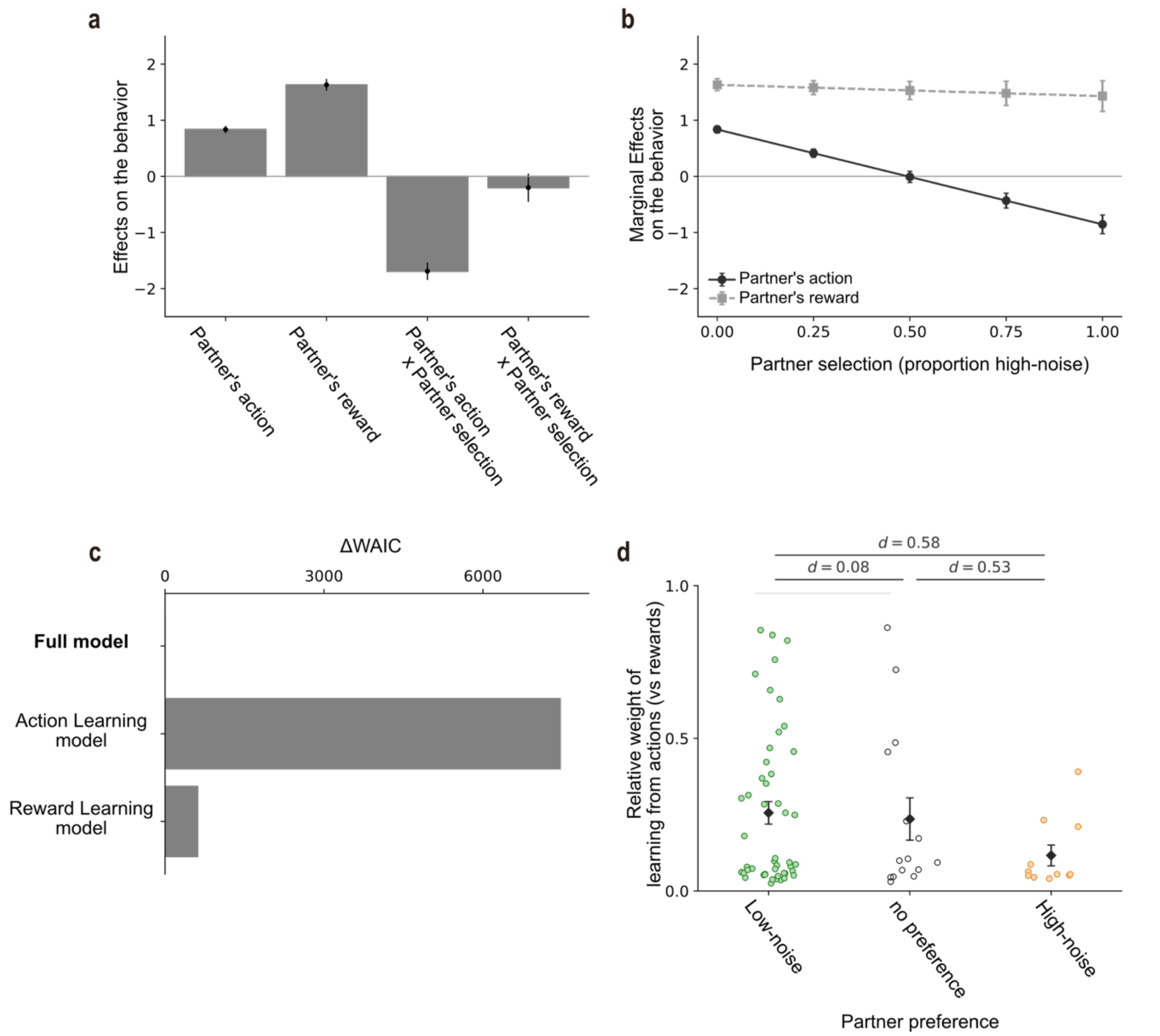

d) Relative weight of learning from actions vs rewards (higher = action) estimated from the full model, grouped by partner preference (low-noise, no-preference, high-noise). Dots are participants; black diamonds show the group mean ± SE. Brackets report Cohen's d for the pairwise contrasts.

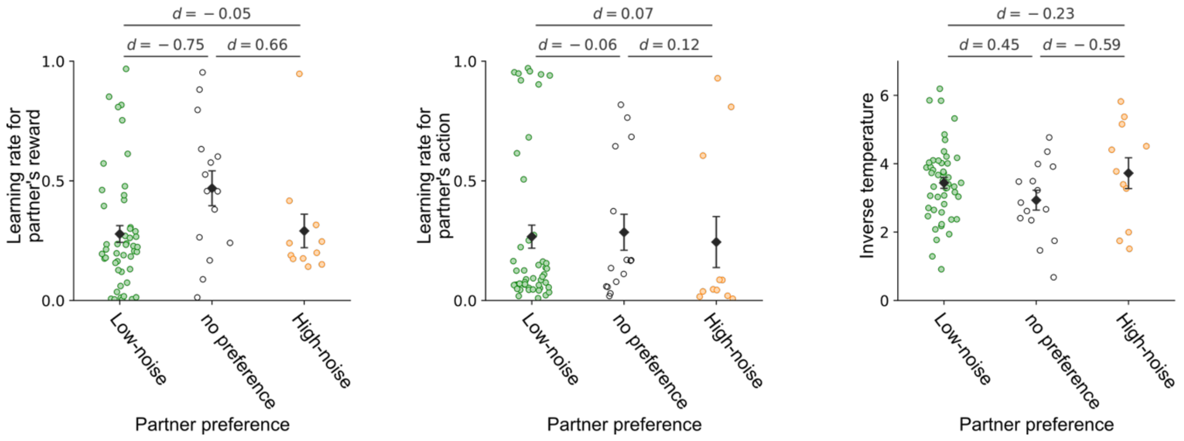

Figure S4: Individual parameter estimates from the Full model, stratified by partner preference (Low-noise, No-preference, High-noise). Left: learning rate for the partner's action (imitation); middle: the learning rate for the partner's reward; right: the inverse

temperature (log scale). Dots show participants; black diamonds indicate group means ± SE. Brackets report Cohen's d for pairwise contrasts.

17. Typo: Figure 3 legend – "explanatory" should be "exploratory".

Reply:

Thank you for pointing out a typo. We have fixed it.

18. The preregistration mentions some exclusion criteria but those are not reported in the Methods. Did any participant meet any of the exclusion criteria?

Reply:

Thank you for pointing this out. In the main experiment, no participants met the preregistered exclusion criteria and all were retained. In the pilot, one participant terminated early (completed only two blocks) and was excluded from the exploratory analyses; all others were retained. We have updated the Methods.

Methods (lines 442-443): "*We did not exclude any participants from the main dataset. For exploratory analyses, we excluded one pilot participant who completed only two of four blocks*."

19. While I appreciate the preregistration effort, I believe the authors should downplay the extent to which the experiment and analyses were preregistered. This is for a few reasons: the preregistration does not report the findings of the pilot study, the observational learning task that was administered after the partner selection task is not described (while I understand the analyses pertaining to this task were exploratory, it seems strange not to have preregistered the task itself and overall protocol prior to data collection), the power analysis is based on equation (1) for the GLMM which is not the analysis that was conducted in the current manuscript (only one predictor of partner selection compared the three), and the power analysis is missing the effect size (which was presumably extracted from the pilot study, but that is not clear). I think it's acceptable to deviate from a preregistration, but this should be more clearly explained.

Reply:

Thank you for raising these important points. We admit that the preregistration did not report the findings of the pilot study. To complement this and improve transparency, we have now added the pilot data results from the preregistered regression analysis and the corresponding power analysis in Tables S1 and S2.

We also apologize for the lack of clarity regarding the observational learning task. This task was included in the preregistration, but its details were provided in the experimental design PDF rather than in the preregistration overview, as its data were not part of the preregistered analyses.

In addition, we have now included the preregistered GLMM results that were missing from the original manuscript.

Finally, to avoid overstating the scope of our preregistration, we have toned down the emphasis on preregistration in the Introduction and Discussion, given that our preregistration did not fully follow best practices.

Results (lines 147-155): "*A mixed-effects logistic regression analysis revealed a significant negative effect of the partner's decision-noise level on partner selection (b = –0.97 ± 0.213, t(55) = –4.557, p < 0.001; Table S1), while controlling for a confounding effect of the spatial position of the partner (i.e., whether the potential partner was displayed on the left or right side of the screen in the Partner Selection task; see Fig. 1b). This effect remained significant (b = –1.06 ± 0.25, t(55) = –4.27, p < 0.001; Fig. 2c), even after additionally controlling for another potentially confounding effect of the presentation order (i.e., whether the potential partner was presented in the first or second Passive Observation task). These results collectively show that most participants preferred to learn from the low-noise partner.*"

Methods: (lines 518-539): "*To address whom participants preferred to learn from during observational learning, we fit mixed-effects logistic regressions with glmer from the lme4 package (version 1.1-35) in R [45, 46]. As preregistered, we analyzed only data from the main experiment (N = 55).*

The dependent variable Y was coded 1 if the left-hand partner was chosen in the Partner Selection task and 0 otherwise. We defined two predictors:

- *X_1: coded +1 if the high-noise partner appeared on the left and −1 if on the right. The coefficient β_1 indexes the effect of partner decision noise on choice (with $\beta_1 > 0$ indicating a preference for the high-noise partner).*
- *X_2: coded +1 if the left-hand partner was presented first in the preceding Passive Observation task and −1 otherwise, controlling for presentation-order bias.*

The intercept captures any overall spatial bias toward choosing the left option (see Fig. 1b).

We estimated two models: Model 1 included X_1; Model 2 additionally included X_2. Participants were treated as random factors, with by-participant random intercepts and random slopes for each fixed effect. We reported point estimates and standard errors of fixed effects, and assessed significance with two-tailed t-tests of coefficients against zero.

Using pilot data, we estimated the effect size for Model 1 (Table S1). Based on this estimate, a bootstrap power analysis [47] indicated that a sample size of N = 55 provides at least 80% power to detect the Model 1 effect at a two-tailed significance level of α = 0.05 (Table S2)."

References

Wilson RC, Geana A, White JM, Ludvig EA, Cohen JD. Humans use directed and random exploration to solve the explore–exploit dilemma. Journal of experimental psychology: General. 2014 Dec;143(6):2074.
