# Supplementary material for: Whom do we prefer to learn from in observational reinforcement learning?
Source: PLoS Comput Biol. 2025 Dec 8;21(12):e1013143. doi: 10.1371/journal.pcbi.1013143 (PMC12697965; doi:10.1371/journal.pcbi.1013143)
Supplement: Attachment — Submitted filename: response_to_reviewer_2nd.pdf [file pcbi.1013143.s012.pdf]

RESPONSE TO THE REVIEWERS' COMMENTS

OVERVIEW

We thank both the reviewers for their thoughtful evaluation of our revision and for the constructive comments provided. We appreciate their time and expertise. Below, we offer a point-by-point response and describe the corresponding revisions made. As the comments pertain exclusively to the Supporting Information, no changes were required in the main manuscript.

Reviewer #2 indicated that all concerns have been addressed; therefore, we provide a detailed response only to Reviewer #1.

Reviewer #1:

Thank you for providing a response revision. Regarding previous major point 3, it would be helpful to also specify the p-value of the correlation, even if this more explorative.

Reply:

We thank the reviewer for this helpful suggestion. We have now added the p-value to the correlation analysis, along with clarifying text.

Supporting information

(line28-30): *The analysis revealed a positive but non-significant relationship between participants' noise levels and their preference for high-noise partners ($r(72) = 0.114 \pm 0.116$, $p = .33$).*
